# Supplementary material for: Analysis of Psychosomatic Disorders According to Age and Sex in a Rural Area: A Population-Based Study
Source: J Pers Med. 2022 Oct 18;12(10):1730. doi: 10.3390/jpm12101730 (PMC9605088; doi:10.3390/jpm12101730)
Supplement: Supplementary file 1 [file jpm-12-01730-s001.zip › jpm-1960079-supplementary.pdf]

Table S1. Diagnostic grouped categories according to ICD-10-ES.

Grouped category	ICD/CIE-10-ES	Diagnostic description
**Depression**	C01F32.0	Major depressive disorder, single episode, mild
	C01F32.1	Major depressive disorder, single episode, moderate
	C01F32.2	Major depressive disorder, single episode, severe without psychotic symptoms
	C01F32.3	Major depressive disorder, single episode, severe with psychotic symptoms
	C01F32.9	Major depressive disorder, single episode, unspecified
	C01F32.89	Other episodes: atypical depression, post schizophrenic depression, single episode of "masked depression"
	C01F33.0	Major depressive disorder, recurrent, mild
	C01F33.1	Major depressive disorder, recurrent, moderate
	C01F33.2	Major depressive disorder, recurrent, severe without psychotic symptoms
	C01F33.3	Major depressive disorder, recurrent, severe with psychotic symptoms
	C01F33.9	Major depressive disorder, recurrent, unspecified
	C01F33.4	Major depressive disorder, recurrent, in remission, no specified
**Persistent mood disorder**	C01F34.0	Cyclothymic disorder
	C01F34.1	Dysthymic disorder
	C01F34.9	Persistent modo disorder [affective], unspecified
	C01F34.89	Other persistent modo disorder [affective]
**Anxiety**	C01F41.0	Panic disorder [episodic paroxysmal anxiety]
	C01F41.1	Generalized anxiety disorder
	C01F41.3	Other mixed anxiety disorders
	C01F41.8	Other anxiety disorders: anxiety-hysteria, mixed anxiety-depressive disorder
	C01F41.9	Anxiety disorder, unspecified
**Stress**	C01F43.0	Acute stress reaction
	C01F43.9	Reaction to stress, severe, unspecified
	C01F43.10	Posttraumatic stress disorder, unspecified
	C01F43.11	Post-traumatic stress disorder, acute
	C01F43.20	Adjustment disorder, unspecified
	C01F43.21	Adjustment disorder with depressed mood
	C01F43.22	Adjustment disorder with anxiety
	C01F43.23	Mixed adjustment disorder, with anxiety and depression
	C01F43.25	Adjustment disorder with mixed disturbance of emotions and conduct
	C01F43.29	Adjustment disorder with other symptoms
**Conversion disorder**	C01F44.4	Conversion disorder with motor symptom or deficit
	C01F44.5	Conversion disorder with seizures or convulsions
	C01F44.9	Dissociative and conversion disorder, unspecified
**Somatoform disorder**	C01F45.0	Somatization disorder
	C01F45.1	Undifferentiated somatoform disorder
	C01F45.8	Other somatoform disorders
	C01F45.9	Somatoform disorder, unspecified
	C01F45.20	Hypochondriacal disorder, unspecified
	C01F45.22	Body dysmorphic disorder
	C01F45.41	Pain disorder exclusively related to psychological factors
	C01F45.42	Pain disorder related to psychological factors
	C01F54	Psychological and behavioural factors associated with somatic functions
**Non-psychotic mental disorder**	C01F48.1	Depersonalization-derealization syndrome
	C01F48.8	Other nonpsychotic mental disorders: neurosis, psychogenic syncope, neurasthenia
	C01F48.9	Nonpsychotic mental disorder, unspecified

**Eating disorder**	C01F50.00	Anorexia nervosa, unspecified
	C01F50.2	Bulimia nervosa
	C01F50.9	Eating disorder, unspecified: atypical anorexia/bulimia, other eating disorder
	C01F50.81	Binge eating disorder
	C01F50.89	Other specified eating disorders: psychogenic loss of appetite, pica in adults
**Sleep disorder**	C01F51.01	Primary insomnia
	C01F51.02	Adjustment insomnia
	C01F51.04	Psychophysiologic insomnia
	C01F51.05	Insomnia due to another mental disorder
	C01F51.09	Other types of insomnia not due to substance or known physiological condition
	C01F51.11	Primary hypersomnia
	C01F51.19	Other types of hypersomnia not due to substance or known physiological
	C01F51.3	condition
		Sleepwalking [somnambulism]
	C01F51.4	Sleep terrors [night terrors]
	C01F51.5	Nightmare disorder
	C01F51.8	Other sleep disorders not due to a substance or known physiological condition
	C01F51.9	Sleep disorder not due to a substance or known physiological condition, unspecified
Signs and symptoms involving emotional state	C01R45.0	Nervousness: nervous tension
	C01R45.1	Restlessness and agitation
	C01R45.2	Unhappiness
	C01R45.3	Demoralization and apathy
	C01R45.4	Irritability and anger
	C01R45.851	Suicidal ideations
	C01R45.6	Violent behaviour
	C01R45.7	State of emotional shock and stress, unspecified
	C01R45.81	Low self-esteem
	C01R45.82	Worries
	C01R45.83	Excessive crying
	C01R45.84	Anhedonia
	C01R45.86	Emotional lability
	C01R45.87	Impulsiveness
	C01R45.89	Other signs and symptoms involving emotional state
Signs and symptoms relating to appearance and behavior	C01R46.0	Very low level of personal hygiene
	C01R46.2	Strange and inexplicable behaviour
	C01R46.3	Overactivity
	C01R46.7	Verbosity and circumstantial detail obscuring reason for contact
	C01R46.89	Other symptoms and signs involving appearance and behaviour
**Malaise and fatigue**	C01R53.1	Asthenia, weakness
	C01R53.81	Other types of malaise: debility, general physical deterioration, malaise
	C01R53.82	Chronic fatigue, unspecified [chronic fatigue syndrome]
	C01R53.83	Other types of fatigue: tiredness, lack of energy, lethargy
Abuse, neglect and other mistreatment, confirmed	C01T74.11	Adult physical abuse, confirmed
	C01T74.11XA	Adult physical abuse, confirmed, initial encounter
	C01T74.12XA	Child physical abuse, confirmed, initial encounter
	C01T74.21XA	Adult sexual abuse, confirmed, initial encounter
	C01T74.21XD	Adult sexual abuse, confirmed, subsequent encounter
	C01T74.22XA	Child sexual abuse, confirmed, initial encounter
	C01T74.22XS	Child sexual abuse, confirmed, sequela
	C01T74.31	Adult psychological abuse, confirmed
	C01T74.31XA	Adult psychological abuse, confirmed, initial encounter
	C01T74.32XA	Child psychological abuse, confirmed, initial encounter
	C01T74.91XA	Unspecified adult maltreatment, confirmed, initial encounter
	C01T74.92XA	Unspecified child maltreatment, confirmed, initial encounter

Abuse, neglect and other mistreatment, suspected	C01T76.11XA	Adult physical abuse, suspected, initial encounter
	C01T76.21XA	Adult sexual abuse, suspected, initial encounter
	C01T76.22	Child sexual abuse, suspected
	C01T76.22XA	Child sexual abuse, suspected, initial encounter
	C01T76.31	Adult psychological abuse, suspected
	C01T76.31XA	Adult psychological abuse, suspected, initial encounter
	C01T76.91XA	Unspecified adult maltreatment, suspected, initial encounter
PR to occupation and unemployment	C01Z56.0	Unemployment, unspecified
	C01Z56.4	Discord with boss and workmates
	C01Z56.5	Uncongenial work environment
	C01Z56.6	Other physical and mental strain related to work
	C01Z56.89	Other problems related to employment
	C01Z56.9	Unspecified problems related to employment
PR to housing and financial circumstances	C01Z59.0	Homelessness
	C01Z59.1	Inadequate housing: unsatisfactory environment, faults and spatial restrictions
	C01Z59.2	Discord with neighbors, lodgers and landlord
	C01Z59.3	Problems related to living in residential institution
	C01Z59.4	Lack of adequate food
	C01Z59.5	Extreme poverty
	C01Z59.6	Low economic income
	C01Z59.7	Insufficient social insurance and welfare support
	C01Z59.8	Other problems related to housing and economic circumstances
	C01Z59.9	Problem related to housing and economic circumstances, unspecified
PR to social environment	C01Z60.0	Problems of adjustment to life-cycle transitions
	C01Z60.2	Problems related to living alone
	C01Z60.3	Acculturation difficulty
	C01Z60.4	Social exclusion and rejection
	C01Z60.8	Other problems related to the social environment
	C01Z60.9	Problem related to social environment, unspecified
**PR to parenting**	C01Z62.0	Inadequate parental control and supervision
	C01Z62.21	Child custody for economic reasons
	C01Z62.22	Institutional upbringing
	C01Z62.6	Inappropriate (excessive) parental pressure
	C01Z62.810	Personal history of physical and sexual abuse in childhood
	C01Z62.811	Personal history of psychological abuse in childhood
	C01Z62.812	Personal history of neglect in childhood
	C01Z62.890	Parent-child estrangement NEC
	C01Z62.891	Sibling rivalry
	C01Z62.898	Other specified problems related to parenting
	C01Z62.9	Problem related to upbringing, unspecified
PR to primary support group, including family circumstances	C01Z63.0	Problems in the relationship with spouse or partner
	C01Z63.1	Problems in the relationship with in-laws
	C01Z63.3	Absence of a family member
	C01Z63.32	Other type of family member absence
	C01Z63.4	Disappearance and death of a family member
	C01Z63.5	Disruption of family by separation and divorce
	C01Z63.6	Dependent relative needing care at home
	C01Z63.72	Alcoholism and drug addiction in the family
	C01Z63.79	Other stressful life events affecting the family and household [health problems within family, ill or disturbed family member, isolated family...]
	C01Z63.8	Other problems related to the primary support group [High expressed emotional level within family, inadequate or distorted communication…]
	C01Z63.9	Problem related to the primary support group [relationship disorder]

Other psychosocial circumstances	C01Z64.0	Problems related to unwanted pregnancy
	C01Z65.0	Conviction in civil and criminal proceedings, without imprisonment
	C01Z65.3	Problems related to other legal circumstances
	C01Z65.8	Other specific problems related to psychosocial circumstances [religious or spiritual problem]
	C01Z65.9	Problem related to unspecified psychosocial circumstances
	C01Z69.81	Encounter for mental health services for victim of other abuse
Counseling related to attitude, behavior and sexual orientation	C01Z70.0	Counseling regarding to sexual attitude
	C01Z70.1	Counseling related to patient's sexual behaviour and orientation [promiscuity, impotence...]
	C01Z70.2	Counseling related to sexual behavior and orientation of third party [partner, child, spouse…]
	C01Z70.3	Counseling related to combined concerns regarding sexual attitude, behaviour and orientation
	C01Z70.8	Other sex counseling [contact for sexual education]
	C01Z70.9	Sex counseling, unspecified
Contact with healthcare services for other types of medical concern	C01Z71.3	Dietary advice and surveillance
	C01Z71.4	Alcohol abuse counseling and surveillance
	C01Z71.41	Alcohol abuse counseling and surveillance of alcoholic
	C01Z71.42	Counseling for family member of alcoholic
	C01Z71.5	Drug abuse counseling and surveillance
	C01Z71.51	Drug abuse counseling and surveillance of drug abuser
	C01Z71.6	Tobacco dependence counseling
	C01Z71.89	Other specific advice
	C01Z71.9	Counseling, unspecified
PR to lifestyle	C01Z72.0	Tobacco use
	C01Z72.3	Lack of physical exercise
	C01Z72.4	Inappropriate diet and eating habits
	C01Z72.51	High risk heterosexual behaviour
	C01Z72.53	High risk bisexual behaviour
	C01Z72.810	Child and adolescent antisocial behaviour
	C01Z72.811	Adult antisocial behaviour
	C01Z72.821	Inadequate sleep hygiene
	C01Z72.89	Other problems related to lifestyle [self-damaging behaviour]
	C01Z72.9	Problem related to lifestyle, unspecified
PR to difficulty in controlling their life	C01Z73.1	Type A behaviour pattern
	C01Z73.2	Lack of relaxation and leisure
	C01Z73.3	Stress, not elsewhere classified [physical and mental tension]
	C01Z73.4	Inadequate social skills, not elsewhere classified
	C01Z73.5	Social role conflict, not elsewhere classified
	C01Z73.6	Limitation of activities due to disability
	C01Z73.9	Problem related to life management difficulty, unspecified
PR to caring for a dependent person	C01Z74	Problems related to the provider of care to the dependent person
	C01Z74.01	Bed confinement status
	C01Z74.09	Other reduced mobility [chair-ridden]
	C01Z74.1	Need for assistance with personal care
	C01Z74.2	Need for assistance at home and no other household member able to render care
	C01Z74.3	Need for ongoing supervision
	C01Z74.8	Other Dependent Care Provider Related Issues
	C01Z74.9	Problem related to care provider dependency, unspecified
